# Sex and Gender Differences in the Effects of Novel Psychoactive Substances

**DOI:** 10.3390/brainsci10090606

**Published:** 2020-09-03

**Authors:** Liana Fattore, Matteo Marti, Rafaela Mostallino, Maria Paola Castelli

**Affiliations:** 1 Institute of Neuroscience-Cagliari, National Research Council (CNR), Cittadella Universitaria, 09042 Monserrato, Cagliari, Italy; 2 Department of Morphology, Surgery and Experimental Medicine, Section of Legal Medicine and LTTA Center, University of Ferrara, 44121 Ferrara, Italy; mto@unife.it; 3 Department of Anti-Drug Policies, Collaborative Center for the Italian National Early Warning System, Presidency of the Council of Ministers, 00187 Rome, Italy; 4 Department of Biomedical Sciences, Division of Neuroscience and Clinical Pharmacology, University of Cagliari, Cittadella Universitaria, 09042 Monserrato, Cagliari, Italy; rafy.mostallino@tiscali.it (R.M.); castelli@unica.it (M.P.C.); 5 National Institute of Neuroscience (INN), University of Cagliari, 09124 Cagliari, Italy; 6 Center of Excellence “Neurobiology of Addiction”, University of Cagliari, 09124 Cagliari, Italy

**Keywords:** NPS, sex/gender differences, cannabinoids, cathinones, phenethylamines, opioids, new synthetic drugs

## Abstract

Sex and gender deeply affect the subjective effects and pharmaco-toxicological responses to drugs. Men are more likely than women to use almost all types of illicit drugs and to present to emergency departments for serious or fatal intoxications. However, women are just as likely as men to develop substance use disorders, and may be more susceptible to craving and relapse. Clinical and preclinical studies have shown important differences between males and females after administration of “classic” drugs of abuse (e.g., Δ9-tetrahydrocannabinol (THC), morphine, cocaine). This scenario has become enormously complicated in the last decade with the overbearing appearance of the new psychoactive substances (NPS) that have emerged as alternatives to regulated drugs. To date, more than 900 NPS have been identified, and can be catalogued in different pharmacological categories including synthetic cannabinoids, synthetic stimulants (cathinones and amphetamine-like), hallucinogenic phenethylamines, synthetic opioids (fentanyls and non-fentanyls), new benzodiazepines and dissociative anesthetics (i.e., methoxetamine and phencyclidine-derivatives). This work collects the little knowledge reached so far on the effects of NPS in male and female animal and human subjects, highlighting how much sex and gender differences in the effects of NPS has yet to be studied and understood.

## 1. Introduction

Men and women differ in terms of physiology and pathophysiology. Male/female differences are important in medicine, and can be responsible for sex-specific clinical manifestations and response to therapies. Sex differences in bioavailability, distribution, metabolism and eliminations of drugs can affect their efficacy and safety and some drugs may be more effective in women than in men, or vice versa [1]. Sex-related differences have been demonstrated for many drugs [2,3,4], including drugs of abuse [5]. Clinical and preclinical studies provided compelling evidence of hormonal- and sex-dependent differences in the wanted and unwanted effects of recreational drugs [6,7,8,9] and in drug sensitivity [10], which may result in a different likelihood of seeking and taking drugs on future occasions and in a different proneness to develop dependence [11]. Socially gendered factors (e.g., social stigma) may also interact with biological factors in modulating drug consumption and the efficacy of therapeutic interventions [12]. According to the last World Drug Report (WDR 2020), drug use is more prevalent among males than females; yet, women are more affected than men by the non-medical use of sedatives and tranquillizers, and substance use disorders are more prevalent in female than in male prisoners [13].

Over the last decade, an incredibly high number of novel psychoactive substances (NPS) have emerged as alternatives to regulated drugs, and new ones are continuously appearing on the internet, social networks and smartphone apps at an incredibly high rate [14]. The NPS market is diverse and dynamic, with the number of NPS rising from 166 by the end of 2009 to 950 substances detected by the end of 2019 [13]. These new drugs are not subjected to clinical trials and information concerning toxicity and specific associated effects is still limited. Yet, animal and human studies showed that NPS are able to elicit not only rewarding and reinforcing effects [15,16,17,18], but also toxic effects of varying severity, at both the peripheral and central levels [19,20], despite an apparent, hazardous perception of safety [21]. Most of them are synthetic cannabinoids and cathinones, new hallucinogen and dissociative drugs or synthetic opioids, these latter representing a major source of social and clinical alarm, due to the numerous fatalities and intoxications associated with their use [22]. NPS represent a growing concern especially for mental health services [23,24], as they have been associated with the risk of violence in patients presenting to acute mental health services [25,26].

The use of NPS is widespread among adolescents, and a nationally representative study enrolling students in 8th to 12th grades across the US showed that boys are at greater risk for using synthetic cannabinoids and synthetic cathinones than girls [27]. Notably, NPS use is increasing in both male and female treatment-seeking opiate-dependent patients as replacement to heroin and other opiates [28], due mostly to practical (e.g., greater availability) and economic rather than pharmacological factors [29]. There is also the possibility that female users may be at risk for being the experimental subjects of immoral drug dealers, i.e., to probe the effects of unknown, experimental synthetic drugs [30].

To date, knowledge on potential sex-dependent effects in the use and abuse of NPS is very scarce. Unfortunately, in many human and clinical studies involving subjects of both sexes, authors did not directly compare females to males, leaving the possibility of the existence of significant sex (animal studies) and gender (clinical studies) differences an open question. Purpose of this review is therefore to examine all animal and clinical studies on NPS involving male and female subjects to check for potential differences or similarities between the two sexes in the prevalence of use and induced drug effects.

## 2. Synthetic Cannabinoids

Synthetic cannabinoid receptors agonists (SCRAs) were initially developed for research purposes, but started being used for recreational purposes in 2004 in Europe and in 2008 in the United States, opening a health and social debate [31]. More than 130 different SCRAs have been detected in recent years, which are currently posing major medical and psychiatric risk worldwide [32]. These compounds typically attract adolescents and young adults due to their affordable cost, perceived legality, and their inability to be detected in urine drug screens [33,34,35]. Compared to Δ9-tetrahydrocannabinol (THC), the principal psychoactive constituent of cannabis, SCRAs are extremely potent full agonists of cannabinoid receptors (CBs), show higher affinity for the CB1 and CB2 receptors [36], and induce longer-lasting and more adverse effects [37,38]. SCRAs misuse has been associated with agitation, anxiety, suicidal ideation, and hallucinations [32,39,40,41], and with generalized seizures and severe toxic injury, including acute kidney injury and myocardial infarction [42]. The long-term or residual effects of SCRAs are almost unknown, but aggression, self-harm and self-mutilation, including self-inflicted burns, have been reported [40,43]. Case reports suggest that SCRAs are able to trigger long-term psychotic symptoms, not only among psychiatric patients, but also in subjects without a previous history of psychosis [44]. Chronic SCRAs users display poorer performance on working memory, cognitive and long-term memory tasks than non-users and recreational cannabis users [45]. Although many new SCRAs show high affinity at both the CB1 and CB2 receptor, it is likely that they exert psychotropic effects, mainly through the activation of CB1 receptors, since mice lacking CB1 receptors, i.e., CB1-knockout mice, do not show the activation of G-protein pathway [46] and increased accumbal dopamine levels [47] after exposure to SCRAs. In support of this notion, CB1 receptor antagonists are able to block SCRA-induced behavioral and reinforcing effects [47], while CB2 receptors seem to be involved more in SCRA-induced neuroinflammation [48].

Animal studies confirmed that SCRAs displayed classic cannabimimetic effects. This was the case for JWH-018 [49,50,51], UR-144 and XLR11 [52], and, more recently, for AB-CHMINACA, AB-PINACA and FUBIMINA [53]. Dose-dependent hypothermia and bradycardia have been reported with JWH-018, UR-144, PB-22, APICA and their fluorinated analogues [54], and with a variety of indazole SCRAs, including AB-PINACA and AB-FUBINACA [55]. Halogenated SCRA derivatives impair motor, sensorimotor, memory and cognitive functions by interfering with hippocampal synaptic transmission [19,56,57]. Fluorinate SCRAs in particular (i.e., 5F-ADBINACA, ABFUBINACA and STS-135) were found to induce a long list of pharmaco-toxicological effects that might explain their detrimental effects in users [58], confirming that fluorination can increase the power and/or effectiveness of these compounds [59]. SCRAs also impair sensorimotor functions [60] and induce a psychostimulant effect, likely by increasing accumbal dopamine release [61], and their combination may potentiate the detrimental effects and increase abuse potential [62].

Sex and gender differences in the rate of cannabis use, prevalence of cannabis use disorders and pharmacological effects have been consistently described by both clinical and animal studies (for comprehensive reviews, see [6,63,64]). Cannabis use is more prevalent among males, but recent data suggest that women are increasing cannabis use at a faster rate than men [65,66], likely due to a decreased perceived risk of cannabis use, legalization and medical marijuana use [67,68,69,70]. A US National Survey of Drug Use and Health has shown that, compared to other classes of NPS, the gender gap in the self-reported use of synthetic cannabinoids is smaller among 12–34 years old users [71], revealing a large consumption of these drugs among female adolescents and young adults.

Women typically report greater sensitivity to the subjective effects of smoked cannabis [72] and to lower doses of orally administered THC [73], and appear more susceptible to developing addiction once use is initiated [74]. However, women (i) are more sensitive to the effects of cannabis and cannabinoids [63], (ii) are less sensitive to the subjective effects of higher doses of THC, probably due to greater tolerance at these doses [73], (iii) may develop problematic cannabis use faster than males and exhibit more intense withdrawal symptoms during abstinence [75].

At present, despite the increased rates of SCRAs abuse, only a limited number of studies have focused on gendered patterns of use and sex-dependent effects. 

Early surveys showed that 11% of USA high school seniors, aged 17–18 years, acknowledge using SCRAs [76], and that males are at greater risk of using them [77]. Similarly, SCRA-intoxicated patients contacting the Texas poison centers in 2010 were 73.9% males, and most frequently reported tachycardia, agitation, drowsiness, vomiting, hallucinations, and nausea [78]. SCRAs are often used with other drugs, such as cannabis and tobacco. In a study on adult marijuana and tobacco users, more than 90% of the population was familiar with SCRA-containing products, and 88% were young adult male and high school graduates aiming to evade drug detection and experience a marijuana-like high [79]. In line with this study, the concomitant use of SCRAs with nicotine and marijuana were found to be more common in male college students than in female students [80]. Further studies and surveys confirmed that SCRAs users are predominantly males who did not attend school, smoke cigarettes, are binge alcohol drinkers and use marijuana daily [81,82,83]. Yet, women seem to initiate use at a significantly younger age [84]. The gender gap in the self-reported use of SCRAs is decreasing among women aged 12–34 years old [71], and occasional emergency room admissions of young women after SCRAs use has been reported [85].

An important sex-dependent difference emerges when looking at side effects, as males required emergency room assistance more often after SCRAs use than after cannabis use (78% and 66%, respectively), while the contrary was observed in female users (22% and 34% after SCRAs and cannabis use, respectively) [86], suggesting that males might be more susceptible to the adverse effects of SCRAs. However, another study seems to suggest the opposite. Using a retrospective chart review for patients admitted to a psychiatric unit, Nia et al. (2019) investigated the relevance of sex in the association of SCRAs use with psychosis and agitation, and found that rates of psychosis were lower in women than in men in the cannabis and control groups, while rates were markedly potentiated but similar in the two sexes (i.e., 79% and 80% in women and men, respectively) in the SCRAs groups, in which females were found to be significantly more agitated than male users [87]. 

Data from preclinical studies confirmed that the effects of THC and early synthetic cannabinoids (e.g., WIN55,212-2) may significantly differ between sexes. For example, female rats were shown to acquire SCRA intravenous self-administration faster than males, stabilize drug intake at higher levels and show greater drug- and cue-induced reinstatement after extinction [88,89], suggesting that females may be more susceptible to their adverse consequences. Female rats seem to be more sensitive than males also to the discriminative stimulus effects of THC, as the THC training dose of 3 mg/kg traditionally used for male rats [90,91,92,93] resulted in being too high for the females [94,95]. In line with this evidence, female rodents are more sensitive to the biphasic effects of cannabinoids on locomotion, and have a greater impairment in spatial learning than males [93,96]. More recently, Borsoi et al. (2019) showed that a single in vivo exposure to WIN55,212-2 differently affects behavior, neuronal and synaptic functions in male and female rats during the pubertal and adulthood periods, and revealed a heightened sensitivity of females, especially during pubescence [97]. Many factors are likely to contribute to the reported male/female differences in cannabinoid-induced effects, including a different CB1 receptor density, distribution or function [98], a sex-specific tonic endocannabinoid signaling [99], a sexual dimorphic activation of specific signaling pathways [100], hormonal influences [101] and possibly pharmacokinetic differences [102,103].

Surprisingly, there is no one animal study specifically designed to examine potential sex-dependent differences in the effects of the last generation of SCRAs. Dedicated studies are urgently needed to unravel the role of sex in the pharmacological and toxicological effects of SCRAs of last generation, in order to help emergency staff to manage intoxication in a more appropriate and sex-tailored manner.

## 3. Synthetic Cathinones

Synthetic cathinones (“bath salts”) are the second largest class of NPS that includes heterogeneous amphetamine-like compounds derived from cathinone, a monoamine alkaloid found in the Khat plant (*Catha edulis*). Like typical psychostimulants (amphetamine-like or cocaine-like compounds), cathinones increase levels of brain monoamines, i.e., dopamine, norepinephrine and serotonin, by acting as monoamine release and/or monoamine reuptake inhibitors [104,105,106,107]. Like synthetic cannabinoids, synthetic cathinones are addictive substances able to induce serious central and peripheral effects, activate the brain reward system and induce abuse-related behaviors [108]. One of the most popular synthetic cathinone of 1st generation found in bath salts, methylenedioxypyrovalerone (MDPV), for example, was found to sustain self-administration behavior and lower the threshold for intracranial self-stimulation in rats, suggesting that it has abuse potential in humans [109]. MDPV also enhances aggressive behavior and locomotion in male mice with greater potency and efficacy than cocaine, and its aggressive and locomotor responses are enhanced after repeated administration, indicating that a sensitization mechanism comes into play [110,111].

Notably, the second generation cathinones pentylone and pentedrone were reported to possess greater abuse liability than first generation cathinone 3,4-methylenedioxymethcathinone (methylone) [112]. Synthetic cathinones were reported to also affect spontaneous and stimulated motor responses [110,113], exert a negative impact on cognitive [114] and memory processes [115] and suppress social behavior in both juvenile and young adult animals [116]. These NPS are frequently used in association with other drugs of abuse, polyconsumption typically resulting in the potentiation of their negative effects [117]. Exposure to these NPS can be very dangerous, and intoxications were found to occur typically through inhalation or ingestion in young (20 years or older) adult males [78]. Compelling evidence has shown that the typical synthetic cathinone users are young males in their twenties and thirties, often experienced drug users, particularly of alcohol and opiates/opioids [118,119]. Males who attend nightclubs are also more likely to use synthetic cathinones than females [120].

Yet, data concerning potential differences in the effects induced by synthetic cathinones in males and females are not fully consistent. In humans, an internet-based cross-sectional survey showed that the pattern of use and self-rated effects of mephedrone, a substituted β-keto amphetamine, are similar in men and women [121]. However, men and women differ for reasons for quitting use of synthetic cathinones, as female users perceive the fear of consequences (i.e., increasing amounts/frequency of use) as more important reasons with respect to the male users’ perceptions [122]. In animals, the cardiovascular effects of the stimulant cathinone (from khat) were similar in males and females [123] while adolescent exposure to methylone was found to induce higher anxiety-related behavior in female than male rats [124]. Similar to (+)-amphetamine- and (+)-methamphetamine [125], cathinone also induced significantly higher increases in motor activity in females than in males, and when administered in combination with caffeine, it significantly elevated body temperature acutely in male but not female rats [126]. Moreover, the duration and intensity of MDPV-induced cardiovascular effects differ among the sexes, lasting significantly longer and being more potent in male than female rats [127]. Moreover, MDPV produced comparable conditioned place preferences in male and female rats, but weaker conditioned taste avoidance in females compared to males [128]. Authors argued that the combination observed in females, i.e., comparable preferences with a weaker avoidance response, might suggest a higher vulnerability to the use and abuse of MDPV than in males.

The existence of contrasting data concerning the effects of a specific compound on males and females does not help to clarify the role of sex in cathinones-induced effects. When looking at the effects induced by the synthetic cathinone α-pyrrolidinopentiophenone (α-PVP, flakka), for example, one study reported that α-PVP intravenous self-administration did not significantly differ between male and female rats [129], while another one showed that males displayed greater α-PVP-induced rewarding (as measured in the place preference paradigm) and aversive effects (i.e., hyperthermia and stereotypies) than females [130]. Hyperthermia is part of the sympathomimetic syndrome caused by synthetic cathinones use, that is associated with acute kidney injury, rhabdomyolysis and, ultimately, death [131,132]. Importantly, it has been recently shown that male and female rats treated once/week with methylone developed tolerance to drug-induced hyperthermia, but females developed tolerance much more rapidly than males while males showed a hyperthermic response over a longer period of time [133]. These studies further confirm sex as a biological variable critical for understanding potential risk factors involved in cathinones use and abuse.

Besides pharmacokinetic factors [134], at present, it is very difficult even to hypothesize the involvement of other factors (e.g., receptor or hormonal male/female differences) in the described sex-dependent differences. Contrary to synthetic cannabinoids, which have been deeper investigated in this sense, no information exists on the possibility that synthetic cathinones may interact with estrogen and testosterone, which could explain (at least in part) the observed male/female differences. The only notion of their impact on sex hormones dates back to 1990, when the natural (not synthetic) cathinone was reported to significantly decrease plasma testosterone levels in rats [135]. Surprisingly, no preclinical or human study has investigated thus far whether these compounds interact with sex hormones and whether the effects of synthetic cathinones in females vary over the estrous/menstrual cycle. The possibility that sex hormones could play a crucial role in the biological effects of cathinones is corroborated by the notion that estrogens and testosterone regulate mRNA expression for dopamine and serotonin receptors in specific brain region [136,137] and contribute to the psychomotor responses of stimulants [136]. Moreover, it has been reported that estrogens could affect cocaine self-administration [138] and reinstatement following extinction [139], and induce greater motivation to seek cocaine [140]. Research in this field is urgently needed for a sex-tailored prevention strategy and the emergency management of intoxications induced by synthetic cathinones.

## 4. Phenethylamines and Tryptamines

Phenethylamines represent a class of NPS with psychoactive and stimulant effects that include amphetamine, methamphetamine, MDMA, ring substituted substances such as the 2C-series compounds, ring substituted amphetamines such as the D-series compound (e.g., DOI, DOC), benzodifurans, such as the fly-series compounds (e.g., bromo-dragonfly) and many others. Tryptamines are a group of monoamine alkaloids with an indole ring structure that includes naturally occurring compounds, such as serotonin and melatonin, but also compound with hallucinogenic properties, such as dimethyltryptamine (DMT), α-methyltryptamine (AMT) and 5-methoxyα-methyltryptamine (5-MeO-AMT). Compounds belonging to these two broad classes of NPS have been found to be common among high-risk youth who reported positive experiences, but also short-term negative health outcomes such as disorientation, hallucinations, nausea, vomiting and diarrhea [141]. Yet, only a few studies examining the prevalence of use or effects induced by phenethylamines included male and female subjects: importantly, all detected subtle, when not significant, sex-dependent differences. Notably, the potential sex-dependent effects of novel recreational tryptamines are fully uninvestigated so far.

As concerns phenethylamines, it was reported that under the effects of 2C-B, verbal fluency did not increase, but speech was more emotional in both male and female healthy recreational users, inter-gender analysis revealing a higher rating in female users [142]. Curiously, two of the female participants cried while performing this task, and one male participant started spontaneously laughing, rendering his speech unintelligible. Authors also reported a reduction in tiredness in men but not in women, suggesting that 2C-B may exert amphetamine-like effects in males and more entactogenic effects in females [142].

Originally synthesized in the early 2000s, the NBOMe drugs are new psychoactive *N*-methoxybenzyl analogues of the 2C-B family of phenethylamines that act as agonists of the serotonin 5-HT2A receptor, thus inducing potent hallucinogen effects [143]. A cross-sectional anonymous online survey investigating the patterns of use of novel drugs reported an incredibly high proportion of males in the NBOMe group that was significantly higher than in the classical hallucinogen group (97.7% vs. 77.5%, respectively) [144]. Further studies support a high prevalence of NBOMe compounds in the young male population [145,146,147,148]. Recently, a preclinical study described for the first time sex-dependent differences in the effects induced by 4-Iodo-2,5-dimethoxy-*N*-(2-methoxybenzyl)phenethylamine (25I-NBOMe), also known as *N*-Bomb, with female and male rats showing, respectively, a higher core temperature and a marked analgesic effect [149], which may account for a gender-specific toxicity, and highlight the possible different pharmacokinetics and pharmacodynamics of NBOMe compounds [150].

Paramethoxymethamphetamine (PMMA) and paramethoxyamphetamine (PMA) belong to a group of methoxylated phenethylamine derivatives that includes the naturally occurring compound mescaline and synthetic compounds, such as methylenedioxymethamphetamine (MDMA) and methylenedioxyamphetamine (MDA). As MDMA, the use of PMMA and PMA seem to be prevalent among the male populations [151,152]. MDMA is known to induce different pharmacological effects in male and female rats, with females showing enhanced sensitivity to its abuse-related behavioral and neurochemical effects [153]. Similarly, MDMA induce more intense physiological (e.g., heart rate) and negative (e.g., psychotic symptoms) effects in women than in men [154].

Regrettably, no study has been conducted so far to assess whether also PMMA and PMA or other newer compounds exert different effects in males and females. Thus, whether the effects induced by the last generations of phenethylamines and tryptamines may be different in males and females is still to be evaluated.

## 5. Synthetic Opioids

Synthetic opioids represent a major threat to public health and have become the fourth large group of substances monitored in 2017, following synthetic cannabinoids, cathinones and phenethylamines [155]. In the USA, the number of deaths involving opioids has more than quadrupled since 1999 [156], in part due to the misuse of prescribed analgesics and the online accessibility to opioids of new synthesis [157]. New synthetic opioids are highly addictive drugs, but have also been associated with various neurological disorders [158], including muscle rigidity, dyskinesia, slow or irregular heart rate, confusion, and anisocoria [159]. Although structurally unrelated to morphine, new synthetic opioids possess similar analgesic and depressant properties [160,161], and are associated with a broad range of side effects, including nasal burn/drip after insufflation, anxiety, sweating, dizziness, nausea, vomiting and disorientation [162]. 

From 2015 to 2017, deaths involving synthetic opioids gradually increased in males and females, the highest rate occurring in males aged 25–44 years [163,164]. However, the mortality gap between men and women is steadily closing [165]. From 2017 to 2018, deaths involving prescription opioids and heroin decreased, while those involving synthetic opioids increased, likely because of illicitly manufactured fentanyl and fentanyl analogs [166]. Indeed, fentanyl is among the most commonly used synthetic opioids, was synthetized in 1960 and approved in 1972 by the Food and Drug Administration (FDA) as anesthetic and for palliative use. Fentanyl analogs and novel synthetic opioids, such as butyrylfentanyl, AH-7921, U-47700 and MT-45 are also widely used nowadays [167].

Similar to the classical opioids (e.g., morphine and heroin), fentanyl binds to opioids receptors, which were among the first neurotransmitter receptors identified biochemically. The existence of multiple classes of opioid receptors in humans was initially suggested by Martin in 1967 [168] and then supported by Gilbert et al. in 1976 [169]. The molecular biology approach led to the cloning of three major classes of opioid receptors: μ-opioid (MOR), δ and κ receptors, which belong to the G-protein coupled receptor (GPCR) family and show a marked structural homology to each other. The gene encoding the MOR, i.e., OPRM1, was isolated and characterized from mouse to human [170,171,172,173], and a remarkable diversity of MOR subtypes was then reported (169). MOR variants have been detected in different regions of the rodent brain, either at mRNA level using RT-PCR, or at protein level, using immunohistochemical or Western blot analysis [174]. Importantly, mRNA expression of these variants was found to be strain-, region- and gender-specific [175,176,177]. Chronic systemic morphine, for example, modifies the spinal expression of mRNA encoding rMOR-1B2 and rMOR-1C1 in a sex-dependent fashion, as in males it resulted in an increased expression of rMOR-1B2 and rMOR-1C1 mRNA, while this effect was completely absent in females [176].

Fentanyl analogs and novel synthetic opioids bind with a greater affinity to MOR, acting potently as agonists of MORs and less actively on δ- and κ-opioid receptor [178,179,180]. Distributed through the peripheral and central nervous system, opioids induce analgesic and rewarding effects, but also adverse effects, such as constipation, hypothermia and respiratory depression [181,182,183,184,185,186].

Clinical research has revealed numerous sex-dependent differences in terms of patterns of use and response to treatment in opioid addicted patients, with women being more likely to use prescription opioids than men, such as Vicodin^®^ and Oxycodone [187], and to benefit from complementary treatment for medical problems during opioid replacement therapy [188]. Sex-dependent differences have also been observed in pain perception and in the response to opioids for pain relief [189,190]. Women typically report more severe pain and a higher prevalence of painful conditions (e.g., irritable bowel syndrome, headache, fibromyalgia, interstitial cystitis), implying higher rates of prescribed opioids, at higher doses and for longer periods than men [191,192]. This might explain, at least in part, why the (mis)use of opioids for medical treatment occurs mostly in women than in men, women being more likely to receive opioids (propoxyphene, codeine, tramadol, morphine and oxycodone) prescriptions than men (54% vs. 46%) [193,194]. As reported in a clinical trial evaluating prescription opioid dependence, women with opioid use disorder reported use for coping with negative emotions and pain [195], while men tended to misuse opioids because of legal and problematic behavioral issues [196].

Contrary to clinical evidence suggesting that men are more sensitive than women to the abuse-related effects of MOR agonists, the majority of preclinical studies suggest the opposite [197,198]. Animal studies have provided a great help in understanding the modulating role of sex in the effects of “classical opioids” (e.g., morphine, heroin) and, to a less extent, of novel synthetic opioids. Male rats, for example, acquire heroin and morphine self-administration less readily than females [199,200], and show a lesser response than females once self-administration is established [201,202]. Female rats display higher responding for heroin and morphine under both fixed-ratio (FR) and progressive-ratio (PR) procedures with respect to males [201,202,203], and show stronger morphine-conditioned place preferences [204,205]. Moreover, although women report significant higher craving for opioids than men [206], few studies have investigated sex as a biological variable in key brain regions engaged during opioid withdrawal [207,208]. Notably, more severe morphine withdrawal symptoms have been observed in male than female rats, although withdrawal symptoms persisted longer in females and were correlated with increased pCREB expression in the ventral tegmental area (VTA) in females, but not in males [209]. As concerns novel synthetic opioids, the modulating role of sex started being investigated only very recently. Specifically, Townsend et al. (2019) have recently evaluated potential sex differences in opioid reinforcement in rats using an intravenous fentanyl vs. food choice procedure. Interestingly, fentanyl sustained a higher responding rate in females under a fixed-ratio schedule of reinforcement, and acted as a more effective reinforcer in females than in males under a progressive schedule [210]. However, when rats were presented with the choice between fentanyl and palatable food, males chose fentanyl more frequently than females, suggesting that a schedule of reinforcements and alternative reinforcers may alter the way the rats allocate their effort.

Besides fentanyl, many other synthetic opioids of more recent synthesis have appeared on the market: the racemic form of MT-45, one of the first designer opioids to achieve some degree of popularity with an in vitro and in vivo potency similar to morphine [211,212], its newly identified fluorinated analogue 2F-MT-45 [213], the highly potent and efficient isotonitazene [214], U-47700 and AH-7921 that caused numerous fatal and non-fatal intoxications [215,216,217], U-50488 and furanyl fentanyl that are frequently encountered in postmortem caseworks [218], U-51754, U-47931E, and methoxyacetylfentanyl [219].

Yet, as for many other NPS, studies focusing on sex-dependent differences in the effects induced by these synthetic opioids of the last generation are completely missing.

## 6. Other NPS

The definition of novel psychoactive substances does not necessarily refer to newly synthetized drugs, but may include failed pharmaceutical products or old patents that are back in vogue as recreational drugs. In this sense, the well-known ketamine is still widely abused worldwide, and represents the reference drug for new dissociative drugs, such as methoxetamine, with which it shares abuse liability and reinforcing effects [220,221], as well as neurological, sensorimotor and cardiorespiratory effects [222]. Gender differences have been reported as concerns drug discontinuation symptoms, with females appearing more susceptible than males to ketamine withdrawal symptoms and adverse effects, such as anxiety, dysphoria, and tremors [223]. Females also displayed a higher rate of concomitant use of hypnotics and alcohol, and a higher severity in cognitive impairment and urinary discomforts [223]. 

Among the over the counter (OTC) medicines, which are delivered without the need for a prescription, loperamide has recently raised concern, due to its euphoric effects and use of alleviating opiate/opioid withdrawal [224]. According to the European Medicines Agency (EMA) EudraVigilance (EV) database, i.e., a high-quality and large-scale pharmacovigilance database, most adverse drug reactions (ADRs) involved adult females that showed abuse/dependence/withdrawal loperamide-related reactions and cardiotoxicity [225]. Another paper from Schifano’s group showed that the so-called “Z-drugs”, i.e., the hypnotics zaleplon, zolpidem and zopiclone proposed on the market as safe substitutes for benzodiazepines, also recently raised clinical concerns due to their recreational use and abuse potential [226]. As for loperamide, the EMA’s EV database revealed that female is the most typically represented gender in Z-drugs-induced ADRs, consisting of misuse/abuse and withdrawal related reactions and overdose-related issues [226]. In light of this and similar reports, stimulating proactive post-marketing surveillance activities would provide a great help in detecting and preventing the misuse potential of prescribed medications [227].

For all the other types of NPS not discussed in the present review, data on gender (human studies) or sex (animal studies) differences are not available at present.

## 7. Conclusions and Future Perspectives

The present study, by summarizing the few data available so far, highlights the existence of a (still limited) number of differences in the behavioral and pharmaco-toxicological responses induced by NPS in male and female subjects. In general, the use of most NPS is prevalent among men than women. Preclinical studies, however, which allow greater control of individual variability (health status, taking other drugs, emotional conditions), have shown that females are more sensitive to the rewarding effects of synthetic cannabinoids and to the anxiety-related effects of synthetic cathinones than males (Table 1). Current knowledge on sex and gender differences in NPS-induced effects is still inadequate, but the need for more studies is supported by the compelling evidence, showing important sex differences in the effects of their referent drugs (e.g., THC, cocaine, amphetamine, morphine). Similar to studies required for monitoring the pharmacological effects of therapeutic drugs, preclinical studies and clinical evaluations are needed to better understand the pharmaco-toxicological effects that NPS cause as a function of sex and gender. Such knowledge, in turn, will allow more effective, sex-tailored interventions to manage acute intoxications and reduce drug use.

## Figures and Tables

**Table 1:** Summary of the evidence available so far describing sex-dependent differences or similarities in the prevalence of use and effects induced by new psychoactive substances (NPS) in males (M) and females (F). The symbol ? indicates the lack of data specifically comparing M vs. F. Abbreviations: 25I-NBOMe: dimethoxy-*N*-(2-methoxybenzyl)phenethylamine; α-PVP: α-pyrrolidinopentiophenone; CPP: conditioned place preference; DD: drug discrimination; IVSA: intravenous self-administration; KET: ketamine; MDPV: methylenedioxypyrovalerone; PPI: pre-pulse inhibition.

	Scras	Synthetic Cathinones	Phenethylamines	Opioids
**Prevalence of use (%)**	M > F [74]	M > F [115,116,117] M = F [118] (mephedrone)	M > F [141,142,143,144,145,148,149]	M > F [160,161] F > M [173,180,181] (prescribed drugs)
**Intoxications (%)**	M > F [75]	M > F [75]	?	M > F [160,161]
**Polydrug use**	M > F [76,77,78,79,80] (nicotine, alcohol, marijuana)	M > F [115,116] (alcohol, opioids)	?	?
**Age of 1st use**	M > F [81]	?	?	?
**Sensitivity to adverse effects**	M > F [83] (general side effects) F > M [84] (agitation, psychosis)	F > M [121] (anxiety, rats) M > F [124] (cardiovascular effects, rats) F > M [130] (tolerance to drug-induced hyperthermia, rats)	F > M [139] (2C-B, emotional verbal fluency) M > F [139] (2C-B, reduction in tiredness) F > M [146] (25I-NBOMe, hyperthermia) M > F [146] (25I-NBOMe, analgesia) M = F [146] (25I-NBOMe, PPI and visual sensorimotor responses)	?
**Sensitivity to rewarding effects** **(animals)**	F > M [85,86,91,92] (IVSA and DD)	M = F [125,126] (MDPV CPP and α-PVP IVSA) M > F [127] (α-PVP CPP)	?	F > M [184] (IVSA) M > F [184] (food choice procedure)
